# Supplementary material for: Convergent and environmentally associated chromatic polymorphism in Bryconops Kner, 1858 (Ostariophysi: Characiformes: Iguanodectidae)
Source: PLoS One. 2024 Feb 15;19(2):e0298170. doi: 10.1371/journal.pone.0298170 (PMC10868817; doi:10.1371/journal.pone.0298170)

Figure S1 - Variation in color of the *B. (Creatochanes)* clade across sample locations. a) *Bryconops (C.) giacopinii* (Manaus - dark Blackwaters); b) *Bryconops (C.) melanurus* (Manaus - light Blackwaters); c) *Bryconops (C.) melanurus* (Coastal - light Blackwaters); d) *Bryconops (C.) aff. affinis* (Xingu - Clearwaters);

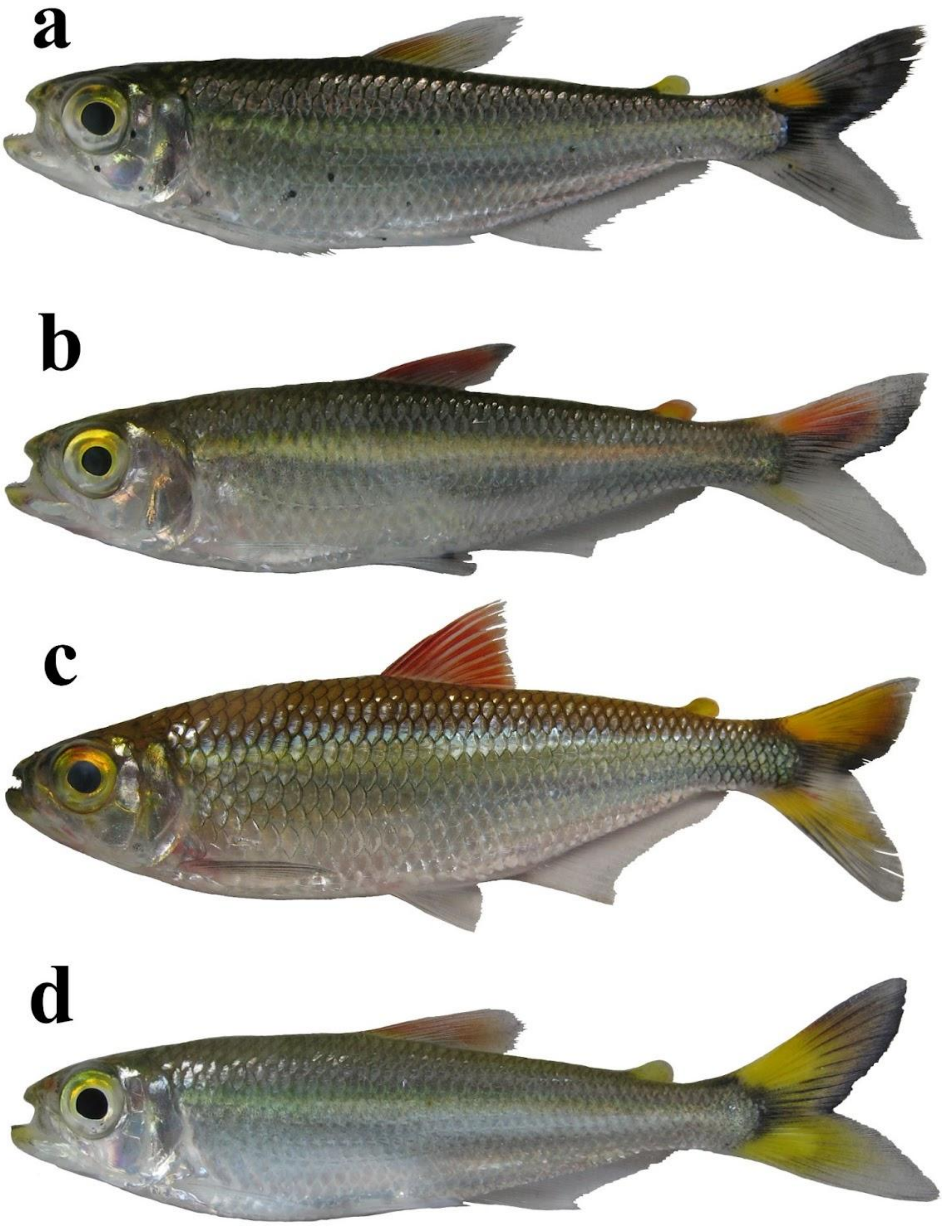

Supplement: S1 Fig — a) Bryconops (C.) giacopinii (Manaus—dark Blackwaters); b) Bryconops (C.) melanurus (Manaus—light Blackwaters); c) Bryconops (C.) melanurus (Coastal—light Blackwaters); d) Bryconops (C.) aff. affinis (Xingu—Clearwaters). (PDF) [file pone.0298170.s001.pdf]
