# Supplementary material for: Convergent and environmentally associated chromatic polymorphism in Bryconops Kner, 1858 (Ostariophysi: Characiformes: Iguanodectidae)
Source: PLoS One. 2024 Feb 15;19(2):e0298170. doi: 10.1371/journal.pone.0298170 (PMC10868817; doi:10.1371/journal.pone.0298170)

Figure S2 - Variation in color of the *B. (Bryconops)* clade across sample locations. a) *Bryconops (B.) caudomaculatus* (Manaus - dark Blackwaters); b) *Bryconops (B.) caudomaculatus* (S Guiana shield - lighter Blackwaters); c) *Bryconops (B.) caudomaculatus* (Coastal - lighter Blackwaters); d) *Bryconops (B.) rheorubrum* (Xingu - Clearwaters and turbid waters).

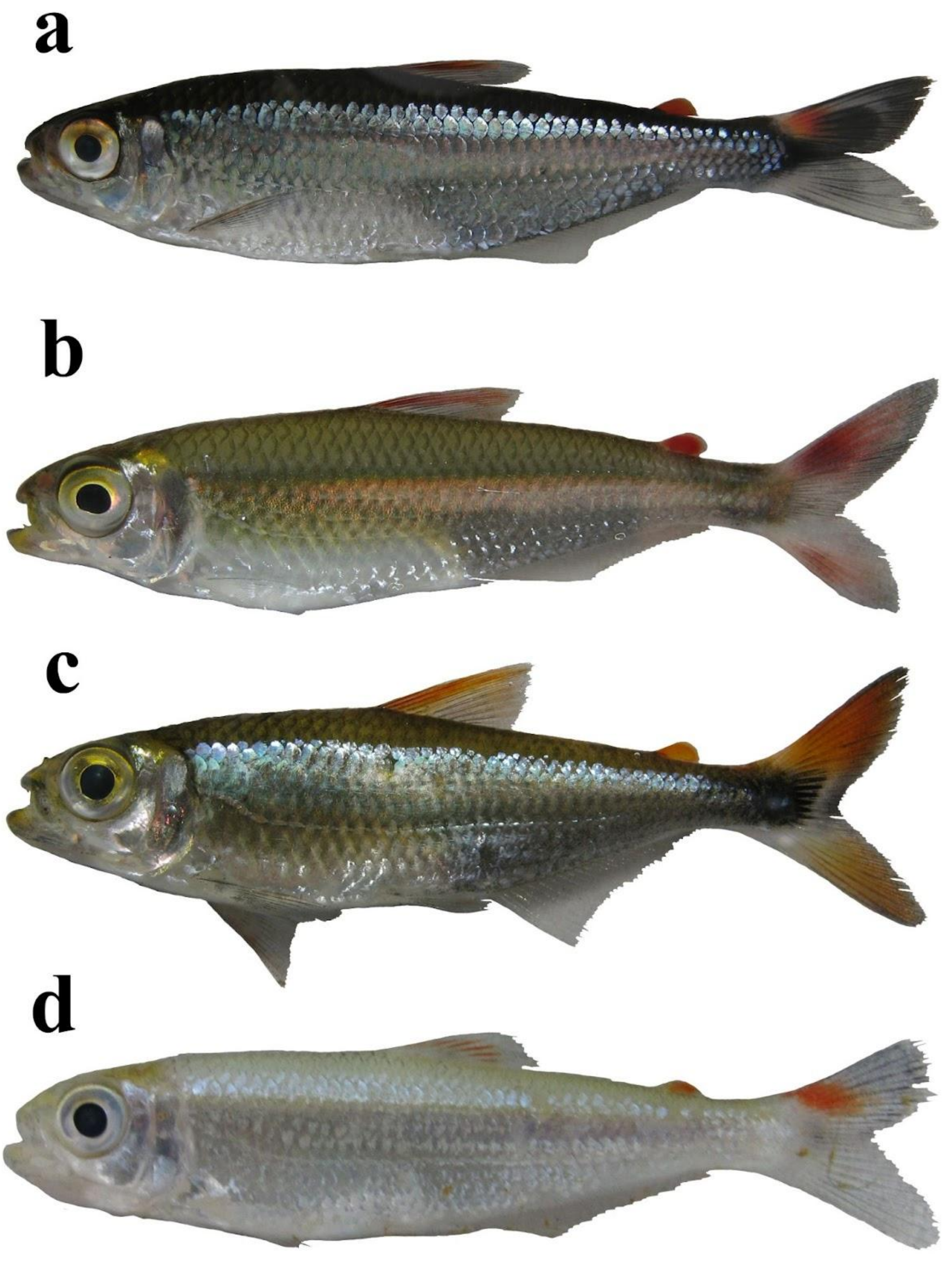

Supplement: S2 Fig — a) Bryconops (B.) caudomaculatus (Manaus—dark Blackwaters); b) Bryconops (B.) caudomaculatus (S Guiana shield—lighter Blackwaters); c) Bryconops (B.) caudomaculatus (Coastal—lighter Blackwaters); d) Bryconops (B.) rheorubrum (Xingu—Clearwaters and turbid waters). (PDF) [file pone.0298170.s002.pdf]
