# Supplementary material for: Convergent and environmentally associated chromatic polymorphism in Bryconops Kner, 1858 (Ostariophysi: Characiformes: Iguanodectidae)
Source: PLoS One. 2024 Feb 15;19(2):e0298170. doi: 10.1371/journal.pone.0298170 (PMC10868817; doi:10.1371/journal.pone.0298170)
Supplement: Attachment — Submitted filename: renamed_73f74.pdf [file pone.0298170.s005.pdf]

Review of Gonçalves et al. "Convergent and environmentally associated plastic chromatic polymorphism in *Bryconops*"

The article aims to test whether color is plastic in *Bryconops* species or if phenotypic variation is related to cladogenetic events. The work is well written and can be a good contribution. However, it presents a problem that needs to be corrected (see below). Therefore, my overall recommendation is either a major revision and resubmit.

The biggest problem has to do with the identity of the species and the consequences for the article's assertions.

The main hypothesis of the work is linked to the supposed wide distribution of two species (*B. caudomaculatus* and *B. melanurus*), which could present polymorphism in the coloration pattern. My comments here do not cast doubt on the hypothesis presented by the authors, but rather on how they use the taxonomy of the group under study.

1) The COI seems like a good tool to demonstrate hidden diversity. However, does COI present sufficient levels of resolution for the recovery of relationships in any of the lineages?

2) Although many authors have mentioned these two taxa as widely distributed, specialists in the group point to the opposite. Chernoff and Machado-Allison (2005), for example, mentioned *B. caudomaculatus* as restricted to the Essequibo River and other similar species occurring in the Amazon basin. Chernoff et al. (1994) mentions that *B. melanurus* occurs only in coastal drainages of the Guianas. Silva-Oliveira (2020) in a broad taxonomic review of the genus, confirms the presence of the true *B. melanurus* only in coastal drainages of the Guianas and recognizes two species complexes (*B. caudomaculatus* and *B. melanurus*) with still underestimated diversity. Probably the authors are working with undescribed species and still without a clear taxonomic delimitation. Although Silva-Oliveira's work has not yet been published, it is important that Gonçalves et al. seek more up-to-date information on the taxonomy of the species they are using as a model.

3) *Bryconops rheoruber* from the supplementary material looks like *B. magoi* or *B. collettei.* According to Silva-Oliveira et al. (2019), *Bryconops rheoruber* presents dorsal fin originates anterior to a vertical through the first pelvic fin ray.

4) Images of some of the species used in the study are missing. As coloration is the focus of the work, all species should be presented.

Other comments are in MS.
