# Supplementary material for: Convergent and environmentally associated chromatic polymorphism in Bryconops Kner, 1858 (Ostariophysi: Characiformes: Iguanodectidae)
Source: PLoS One. 2024 Feb 15;19(2):e0298170. doi: 10.1371/journal.pone.0298170 (PMC10868817; doi:10.1371/journal.pone.0298170)
Supplement: Attachment — Submitted filename: Bryc_POne_Response to Reviwers.doc [file pone.0298170.s007.doc]

Journal requirements:

When submitting your revision, we need you to address these additional requirements.

1. Please ensure that your manuscript meets PLOS ONE's style requirements, including those for file naming. The PLOS ONE style templates can be found at

<https://journals.plos.org/plosone/s/file?id=wjVg/PLOSOne_formatting_sample_main_body.pdf> and

<https://journals.plos.org/plosone/s/file?id=ba62/PLOSOne_formatting_sample_title_authors_affiliations.pdf>

**REPLY –** The changes were made in both the main body of manuscript and authors affiliation.

2. Thank you for stating the following financial disclosure:

“The authors thank CAPES (Coordenação de Aperfeiçoamento de Pessoal de Nível Superior - Brasil - Finance code 001) for studentships for ASG, SCS, ACGR, SMG and DFJS through the postgraduate programmes PPGEAP and PPGBA), and research funding from CNPq (Conselho Nacional de Desenvolvimento Científico e Tecnológico) through the Brazilian Barcode of Life (BrBOL, process 64953/2010-5) and Research Productivity Grant (process 313834/2021-0), FAPESPA (Fundação Amazônia de Amparo a Estudos e Pesquisas) Programa Primeiros Projetos grant 011/2009, FAPERGS (Fundação de Amparo à Pesquisa do Estado do Rio Grande do Sul) ARD/ARC process 72550.751.48979, Vale (Centro de Triagem de Invertebrados contract R100603.CT.02), Hydro through the Biodiversity Research Consortium Brazil-Norway (BRC project 16/19) and National Science Foundation (NSF DEB-1146374).

NO”

Please state what role the funders took in the study.  If the funders had no role, please state: "The funders had no role in study design, data collection and analysis, decision to publish, or preparation of the manuscript."

If this statement is not correct you must amend it as needed.

Please include this amended Role of Funder statement in your cover letter; we will change the online submission form on your behalf.

**REPLY** – The funders had no role in study design, data collection and analysis, decision to publish, or preparation of the manuscript.

3. We note that you have stated that you will provide repository information for your data at acceptance. Should your manuscript be accepted for publication, we will hold it until you provide the relevant accession numbers or DOIs necessary to access your data. If you wish to make changes to your Data Availability statement, please describe these changes in your cover letter and we will update your Data Availability statement to reflect the information you provide.

**REPLY –** The accession numbers are already provided in Table S1.

4. We note that Figures 1 and 2 in your submission contain [map/satellite] images which may be copyrighted. All PLOS content is published under the Creative Commons Attribution License (CC BY 4.0), which means that the manuscript, images, and Supporting Information files will be freely available online, and any third party is permitted to access, download, copy, distribute, and use these materials in any way, even commercially, with proper attribution. For these reasons, we cannot publish previously copyrighted maps or satellite images created using proprietary data, such as Google software (Google Maps, Street View, and Earth). For more information, see our copyright guidelines: <http://journals.plos.org/plosone/s/licenses-and-copyright>.

We require you to either (1) present written permission from the copyright holder to publish these figures specifically under the CC BY 4.0 license, or (2) remove the figures from your submission:

1. You may seek permission from the original copyright holder of Figures 1 and 2 to publish the content specifically under the CC BY 4.0 license.

We recommend that you contact the original copyright holder with the Content Permission Form (<http://journals.plos.org/plosone/s/file?id=7c09/content-permission-form.pdf>) and the following text:

“I request permission for the open-access journal PLOS ONE to publish XXX under the Creative Commons Attribution License (CCAL) CC BY 4.0 (<http://creativecommons.org/licenses/by/4.0/>). Please be aware that this license allows unrestricted use and distribution, even commercially, by third parties. Please reply and provide explicit written permission to publish XXX under a CC BY license and complete the attached form.”

Please upload the completed Content Permission Form or other proof of granted permissions as an "Other" file with your submission.

In the figure caption of the copyrighted figure, please include the following text: “Reprinted from [ref] under a CC BY license, with permission from [name of publisher], original copyright [original copyright year].”

2. If you are unable to obtain permission from the original copyright holder to publish these figures under the CC BY 4.0 license or if the copyright holder’s requirements are incompatible with the CC BY 4.0 license, please either i) remove the figure or ii) supply a replacement figure that complies with the CC BY 4.0 license. Please check copyright information on all replacement figures and update the figure caption with source information. If applicable, please specify in the figure caption text when a figure is similar but not identical to the original image and is therefore for illustrative purposes only.

The following resources for replacing copyrighted map figures may be helpful:

USGS National Map Viewer (public domain): <http://viewer.nationalmap.gov/viewer/>

The Gateway to Astronaut Photography of Earth (public domain): <http://eol.jsc.nasa.gov/sseop/clickmap/>

Maps at the CIA (public domain): <https://www.cia.gov/library/publications/the-world-factbook/index.html> and <https://www.cia.gov/library/publications/cia-maps-publications/index.html>

NASA Earth Observatory (public domain): <http://earthobservatory.nasa.gov/>

Landsat: <http://landsat.visibleearth.nasa.gov/>

USGS EROS (Earth Resources Observatory and Science (EROS) Center) (public domain): [http://eros.usgs.gov/#](http://eros.usgs.gov/)

Natural Earth (public domain): <http://www.naturalearthdata.com/>

**REPLY –** The layers in both maps were made using files from IBGE (<https://www.ibge.gov.br/geociencias/downloads-geociencias.html>) - for hydoragraphy, and regional maps - that states that all files are public, and Natural Earth (<http://www.naturalearthdata.com/>) for South America landmass.

5. Please remove your figures from within your manuscript file, leaving only the individual TIFF/EPS image files, uploaded separately. These will be automatically included in the reviewers’ PDF.

**REPLY** – Figures removed from text, leaved only the legends in place.

Review Comments to the Author

Reviewer #1:Review of Gonçalves et al. “Convergent and environmentally associated plastic chromatic polymorphism in Bryconops”

The article aims to test whether color is plastic in Bryconops species or if phenotypic variation is related to cladogenetic events. The work is well written and can be a good contribution. However, it presents a problem that needs to be corrected (see below). Therefore, my overall recommendation is either a major revision and resubmit.

The biggest problem has to do with the identity of the species and the consequences for the article's assertions.

The main hypothesis of the work is linked to the supposed wide distribution of two species (B. caudomaculatus and B. melanurus), which could present polymorphism in the coloration pattern. My comments here do not cast doubt on the hypothesis presented by the authors, but rather on how they use the taxonomy of the group under study.

1) The COI seems like a good tool to demonstrate hidden diversity. However, does COI present sufficient levels of resolution for the recovery of relationships in any of the lineages?

**REPLY** – COI data has previously been shown to provide a reasonable level of information for resolving recent divergence in many fish groups from South America. furthermore, as the analysis depends on the strength of the formation of clades and this is determined by the support of nodes within the phylogeny (which in turn depends on the information available in any given dataset) we can consider that the dataset is informative as there is a clear separation of clades with generally good node support and consistent with the deeper level separation of subgenera as described in morphological studies.

2) Although many authors have mentioned these two taxa as widely distributed, specialists in the group point to the opposite. Chernoff and Machado-Allison (2005), for example, mentioned B. caudomaculatus as restricted to the Essequibo River and other similar species occurring in the Amazon basin. Chernoff et al. (1994) mentions that B. melanurus occurs only in coastal drainages of the Guianas. Silva-Oliveira (2020) in a broad taxonomic review of the genus, confirms the presence of the true B. melanurus only in coastal drainages of the Guianas and recognizes two species complexes (B. caudomaculatus and B. melanurus) with still underestimated diversity. Probably the authors are working with undescribed species and still without a clear taxonomic delimitation. Although Silva-Oliveira’s work has not yet been published, it is important that Gonçalves et al. seek more up-to-date information on the taxonomy of the species they are using as a model.

**REPLY** – Whether or not the species are described does not influence the interpretation of convergence (though does impact discussions of plasticity - see point 2 of reviewer 2). a fully resolved taxonomy of all samples is an unrealistic target for inclusion in this work in the near future as there are many unsampled regions and classifications are always based on current available published information and valid species. We use the taxonomic classifiers aff and cf to recognize discrepancies and variants where possible considering both the morphological identifications made and molecular lineages identified.

3) Bryconops rheoruber from the supplementary material looks like B. magoi or B. collettei. According to Silva-Oliveira et al. (2019), Bryconops rheoruber presents dorsal fin originates anterior to a vertical through the first pelvic fin ray.

**REPLY** - The identification was made according to the original description of *B. rheoruber* in Silva-Oliveira et al. 2019 ([doi.org/10.1111/jfb.14445](https://doi.org/10.1111/jfb.14445)).

4) Images of some of the species used in the study are missing. As coloration is the focus of the work, all species should be presented.

**REPLY** – Unfortunately we don’t have all the photos within Belém Group, although we asked them for the others authors, we don’t have answers. The photos in Figures S1 and S2 represents all the species we have pictures (we have more pictures of the same species that we judge would only pollute and make difficult to understand if we put all of them together).

Other comments are in MS.

**REPLY** – We have altered the text in accord with some of your suggestions. About the classification of water types in Table 1, we describe it in the last paragraph of methodology (lines 225-229), we couldn’t inform the drainage in this table, but all sample locations are provided in Table S1.

Reviewer #2: Gonçalves et al present an interesting study examining whether color patterns in the iguanodectid fish genus Brycanops exhibits convergence or has a strong phylogenetic signal and thus can be used as a character for taxonomic delimitation. The authors score colors from specimens collected from different water types and use sequences of COI to create a phylogeny. Species partitioning methods, tests of phylogenetic signal, and a test of convergence are then employed to test the hypothesis. Although a more robust phylogeny using more genes or genomic methods and the inclusion of more specimens would have been good, the methods seem robust to make the point the authors advocate. The point that phenotypic similarity of ecologically important traits may often be the result of convergence and may complicate phylogenetic inference or species delimitation using these traits is also an important one to make.

I do have some points that I think should be addressed, which are presented below.

-I was disappointed that there was not a figure in the paper itself with the characters scored that showed the traits and the color variation. Two figures are presented in the supplemental files but given how central they are to understanding the results, I strongly encourage the authors to combine these into one figure and include them in the manuscript itself. Putting them in the supplemental files just makes things more difficult for readers. In addition, PLoS one will have a general readership and many may have difficulty understanding the traits without seeing them.

-The abstract and some parts of the paper are written in a way that makes it seem like convergence and plasticity are synonyms, which is very misleading. For example, from the abstract (lines 26-28): “…To test whether color is plastic in those species or if phenotypic variation is related to cladogenetic events…“. What is being tested is whether the trait is related to the phylogenetic relationships of the species or not. However, there is no way of knowing if it is phenotypically plastic, as the authors indicate themselves in the Discussion. Phenotypic plasticity refers to non-genetic changes in the phenotype, that is, changes in gene expression patterns that result in different phenotypes in fish that are identical for the underlying alleles. Convergence generally refers to convergent evolution, the independent evolution of similar phenotypic traits, often related to adaptation to similar environmental characteristics. They are different things. The text should be reviewed for how this is phrased, especially the abstract and introduction (although there may be problems in other sections). The authors should indicate that they are testing for phylogenetic independents and conducting a test of phenotypic convergence, with the plasticity text saved for the discussion, where they indicate possible causes and that it is not possible to distinguish between phenotypic plasticity and evolutionary convergence with these data.

**REPLY** – Thanks, we already changed the text.

Lines 55-57: Oliveira et al. (2011) is listed as the latest phylogenetic hypothesis of the Characiformes, but this is a phylogeny of the Characidae and there are more recent phylogenies of Characids (e.g., Mirande 2018). This should be reworded.

**REPLY** – It was reviewed, we already had the Mirande citation in the next paragraph, thanks.

-Lines 102-104: I assumed that saying that traits are phylogenetically independent is the same as saying that they are evolving according to Brownian motion. If they are phylogenetically dependent, they would be more similar than null expectations under Brownian motion, right? What am I missing? Can this be explained more carefully since the topic can be confusing.

**REPLY** - Assuming a trait as a phylogenetically independent is the same as saying that it is not evolving in accord to neutral assumption, not evolving accord to Brownian Motion implied by some of the tests. If so, there is a force (with strength, theoretically, proportional to the variation in the indices) influencing the phenotype, this could be a result from sexual selection (as you suggested), from difference in predation due to mimetism, from dietary origin, or a combination of those factors. In this work we report that such possibility exists, but we would need more samples from the same localities to test if there is a deviation from Hardy-Weinberg equilibrium, considering we have the right genetic markers for it.

-Line 118: Indicate how many specimens from how many sites are included. The info is in the tables but would be good to make it explicit up front and not make the reader have to count.

**REPLY** – Changed the text.

-Lines 144-147: How was color standardized, was a color scale included in the photographs? If not, lighting under which the pictures were taken may interfere with the ability of the authors to score the colors accurately. This should be addressed in the text.

**REPLY** - A color scale (Spyder Checkr) was originally included in some but not all photographs and was not available for some collections. Given our experience that the variation observable was not quantifiable directly using digital color coding but that within the samples we did have color standards the effects of light intensity/shade did not alter a simple classification between yellow, orange, and red coloration, we opted to continue the analysis considering this gross classification that could be reliably separated and tested

-Table 1: Please indicate how many individuals were collected from each water type. This information can easily be included given the structure of the table and will be helpful for the reader.

**REPLY** – Changed.

-Line 200 should be ABGD, not ABDG. Please check other parts of the manuscript for this typo.

**REPLY** – Thanks for showing the error, it is already changed.

-Lines 200-202, the ABGD method has several parameters one sets. What were the other values used and why? If the other methods have options for other parameters, these should be indicated as well. Otherwise, make sure to note that the defaults were used.

**REPLY** – The others setting not commented were not altered, changed the text to cover this.

-Line 211 also talks about scoring colors, so addressing how color was standardized could be done here.

**REPLY** – Color standard was replied here and we changed the text to cover for this possible doubt of the readers.

-Line 262: Has MOTU been defined already? It should be defined before it is used.

**REPLY** – Sorry, we altered the text.

-Line 295: add a comma after “Brownian Motion models”.

**REPLY** – Thanks, we altered the text.

-Lines 355-356: What if the water color leads to similar sexual preferences because similarities of how the fish colors contrast with particular water colors? Maybe some colors patterns stand out more in some water types. That could lead to a correlation between water types and sexual preferences for particular fin color patterns.

**REPLY** - We agree that it could be a possibility, however, for the lack of sexual dimorphism between sexes, we should think that this process occurs in both sexes? Or that it could be caused by a founder effect (small effective size and strong sexual selection force for the color and now the entire population has the characteristic)? To respond both hypotheses we would need more samples from the same localities and investigate both autossomic and sexual markers.
