# Supplementary material for: Convergent and environmentally associated chromatic polymorphism in Bryconops Kner, 1858 (Ostariophysi: Characiformes: Iguanodectidae)
Source: PLoS One. 2024 Feb 15;19(2):e0298170. doi: 10.1371/journal.pone.0298170 (PMC10868817; doi:10.1371/journal.pone.0298170)
Supplement: Attachment — Submitted filename: Bryc_POne_Response to Reviwers2.doc [file pone.0298170.s008.doc]

Additional Editor Comments:

The page and line numbers refer to the clean version of the manuscript in revision 1.

-For the title: I do not think that you have shown whether the pattern is the result of plasticity (non-genetic phenotypic change), so it seems misleading to include plasticity in the title. I think you can just remove the plastic from the title and leave it as: “Convergent and environmentally associated chromatic polymorphism in Bryconops Kner, 1858 (Ostariophysi: Characiformes: Iguanodectidae)”. The “environmentally associated” leaves the possibility of plasticity without actually using the word without showing that it is plasticity.

**REPLY –** Changed as requested. We had meant to remove this word in the previous revision but it slipped through.

-Line 27: Add a comma “,” after cladogenetic events.

**REPLY – done – now at line 28**

-Lines 34-37: For the last line of the abstract, it could be plasticity or convergent evolution. Either could cause problems for grouping or identifying species. I think you need to add the possibility of convergent evolution to the sentence and suggest modifying it to:

“This means that simple color characters cannot necessarily be relied upon for taxonomic revisions of the genus as local phenotypic variants may represent environmentally determined plasticity or convergent evolution. Further studies are required to determine the validity of these characters.”

**REPLY – we have followed the suggestion**

-Lines 53-57: The modification to include “actually” does not work very well the way the word is used here. Although it is a long sentence, I suggest changing to:

“The genus includes Cis-andean small to medium sized tetras widely distributed in the Orinoco, Amazonas, Tocantins-Araguaia, Paraná-Paraguai, São Francisco rivers and several coastal basins draining from the Brazilian and Guiana shields [10-12]. Bryconops is currently included in the family Iguanodectidae along with Iguanodectes Cope, 1872 and Piabucus Oken, 1817 [13-14].”

**REPLY – we have followed the suggestion**

-Line 80: Replace “demand” with “need”.

**REPLY – done**

-Lines 112-113: Change to: “to test if such variation is independent of the evolutionary history and is correlated with water color from the specimen’s localities.”

**REPLY – we have changed as requested**

**Lines 124-128 in the current revision. We improved the text to clarify the new sampling targeted for this study and the preexisting samples that were possible to include because of available and informative metadata.**

-Line 148: Divide into two sentences. So: “photos, but was not available for all specimens. To avoid possible bias in using digital”

**REPLY – done (also corrected “spectre” to “spectrum”)**

-Table 1 as currently formatted is confusing because the records are not clearly separated between species. Please make the separation between species easier to see.

**REPLY – we have added ditto marks to clarify the same species as above and justified the total counts to make this clearer.**

-Line 203: Modify to: “(k80) model with TS/TV = 2.0. Default settings were used for the remaining parameters. bPTP was run via”.

**REPLY – changed as requested.**

-Line 211: Add a comma “,” after “For new samples,”

**REPLY** – done

-Table 2: I assume that the lower and upper bounds and P are for the Wheatsheaf test. Please indicate so in the caption o there is no doubt. Also, not that for the Dorsal fin melanic patch, the numbers are on two different lines. The same happens for the two last traits. I assume this is not intentional and should be corrected.

**REPLY – the table has been corrected as requested.**

-Table 2: Why were the P values removed from Abouheif’s C mean and Pagel’s test? Why were Moran’s I and Blomberg K removed? In the previous versions, these tests indicated strong significance. Now there is no indication of this. The actual numerical values have also changed. In the new version, only the color of the dorsal fin has a P value of less than 0.05. This needs to be explained carefully to ensure that the results still hold true since this involves the key component of the analysis and interpretation of convergence in these color traits.

**REPLY** – The first version of the table was a provisional copy, made while we were creating the code for the analysis. The indices mentioned should not have got into the final manuscript and, while nobody commented on it, we feel that the right choice was to correct the table with the final data and the final code. As the data used were partial, some results differed slightly, but the signal didn’t change for the results and the traits that showed signals of independence, continued to show the same signals.

Regarding the logic of the removal of Moran’s I and Blomberg K, we follow the suggestions of Münkemüller et al. 2012 (<https://doi.org/10.1111/j.2041-210X.2012.00196.x>). In this work Abouheif’s C_mean_ and Pagel’s λ presented the best overall performance, while Blomberg K presents high sensitivity to noise in phylogenetic signal, to the point where it can point to divergent conclusions from the others methods. For Moran’s I, while both Moran and Abouheif tests are dependent on sample size, Moran’s I also showed more sensitivity to branch length and polytomies. The higher sensitivity of both Moran and Blomberg tests can be a problem with the use of Bayesian inferred trees, especially for the reproduction of the results with the same dataset.

Both, Abouheif’s C_mean_ and Pagel’s λ, results can vary from 0 to 1, if the result stays closer to 0, it shows a higher independence from the phylogenies, while we can’t compare both values quantitatively, they show a trend of the independence for each trait analyzed. The most important is the value, not the significance. On the contrary, for the Wheatsheaf the most important is the significance of the results, it is what shows that there is convergence between a trait and an external factor. If you see the tables again, you can confirm that the result present in the table 2 is the same as in the old table 3 (old tables 2 and 3 were merged).

-I would have liked to see figures S1 and S2 merged into one figure and included in the main manuscript. I think the pictures are beautiful and help the non-specialist understand the color characters better. However, I will not insist if the authors prefer not to make this modification for some reason. Just make sure to update the figure numbers if you do.

**REPLY** – We feel that it would create more visual “noise” in the main manuscript rather than improve the understanding.
